# Variations of root and canal morphology of mandibular second molars in Chinese individuals: a cone-beam computed tomography study

**DOI:** 10.1186/s12903-022-02299-8

**Published:** 2022-07-05

**Authors:** Lin Yang, Jingchao Han, Qibao Wang, Zhuoran Wang, Xijiao Yu, Yi Du

**Affiliations:** 1 Department of Endodontics, Central Laboratory of Jinan Stomatological Hospital, Jinan Key Laboratory of Oral Tissue Regeneration, Jinan, Shandong China; 2 Department of Radiology, Jinan Stomatological Hospital, Jinan, Shandong China

**Keywords:** Anatomy, Cone-beam computed tomography, Molars

## Abstract

**Background:**

The mandibular second molars demonstrate variations on root and canal morphology. The aim of this study was to investigate all the root canal morphology of mandibular second molars and analyze the morphological variations in patients by gender and age in a Chinese population use CBCT imaging.

**Methods:**

Cone-beam computed tomographic images of 1200 bilateral mandibular second molars were obtained from 600 patients (300 females and 300 males) who required a preoperative assessment for implant surgery, surgical removal of impacted teeth, orthodontic treatment, surgery of maxillofacial tumour and cysts or LeFort I osteotomy. CBCT images were divided into 5 groups according to age: “15–24 years”, “25–34 years”, “35–44 years”, “45–54 years” and “≥ 55 years”; and 2 groups by gender: “females” and “males”. The following information were recorded: the number of roots and canals and their morphology, the frequency and configuration of C-shaped canals by gender, age and position (left and right). The chi-square test was used to analyse differences between groups. *P* value of < 0.05 was considered statistically significant.

**Results:**

Of the 1200 teeth, 61% had two separate roots located mesiodistally, 35.6% had one C-shaped root. The 45.3% teeth had three canals in two-rooted mandibular second molars. The mesial root showed a Vertucci type II configuration in 28.9% cases followed by type IV(24.4%). While the distal root showed a significant higher prevalence of type I configuration in 95.6%. In the examined 1200 teeth, 430 teeth (35.8%) had C-shaped root canals. The prevalence of C-shaped root canal systems was significantly higher in females (42.5%) than in males (29.1%) (*P* = 0.000), and did not differ with age (*P* = 0.126). The 80.4% C-shaped canals were bilateral (*P* = 0.000) and did not differ with side (left and right) (*P* = 0.758).

**Conclusions:**

The most commonly observed root morphology for the mandibular second molars was 2 separate roots with three canals.The prevalence of C-shaped root canal is 35.8% and is more higher in females than in males.

## Background

A successful root canal therapy include complete debridement, disinfection and obturation [1]. Incomplete instrumentation followed by incorrect obturation, untreated or missed canals that contain microorganisms can cause endodontic treatment failure [2]. Therefore, recognition of the root anatomy and the internal complex 3- dimensional root canal morphology is essential for clinicians and benefit to long-term success [3].

The majority of mandibular second molars had two separate roots and three canals (2 in mesial root and 1 in distal root) [4–9], and the Vertucci’s type IV (2-2) or Type II (2-1) canal configuration [10] was the most prevalent in mesial roots while the type I was the most common canal configuration in distal roots. Moreover, the rare 3 canals also have been discovered in the mesial root of two -rooted mandibular second molars which represented a challenge for root canal treatment [11, 12]. First described by Cooke et al. [13], the prevalence of C-shaped mandibular second molars had been reported varied in various populations among the worldwide (2.7–45.5%) [14]. China had a significantly higher prevalence of 44.0% than any other region. In addition, women presented a significantly higher prevalence of C-shaped canals than men [14–16].

There were reports on the root canal morphology of mandibular second molars in Chinese population after reviewing the literature using the PubMed Database (National Library of Medicine). In studies by Wang et al. [17], Zheng et al. [18] and Ren et al. [19], they reported the information of C-shaped root canal system. In another study by R. Zhang et al. [7], they reported the distributions of variants and C-shaped canals in the mandibular second molars. Although these studies improved valuable data on the root canal anatomy, they have limitations in some ways. For example, (i) small sample sizes [7, 19]; (ii) limited groups of teeth without age and gender-the root canal system configuration changes over a lifetime [10], age related morphological variations may present a challenge to the clinician as they increase the difficulty of treatment [20]; (iii) incompleted information of the non-C-shaped teeth; (iiii) a restricted method: using radiography and clinical examination under microscope might not be able to represent the 3-dimensional images of the root canal anatomy especially C-shaped configurations indeed vary along the root length [16]. Compared with the periapical radiography, CBCT imaging can provide 3-dimensional images from the axial, sagittal, and coronal sections as well as eliminate the superimposition of surrounding structures [21]. As a noninvasive tool, CBCT scanning has the capacity to detect the root canal system as accurately as the staining and clearing techniques used to study extracted teeth [22].

In studies by Zheng et al. [18] and Ren et al. [19], they reported the information of C-shaped root canal system by gender and age while other root canal morphologies were not referred. Therefore, the aim of this study was to use CBCT imaging to investigate all the root canal morphology (C-shaped and non-C-shaped) of mandibular second molars and analyze the morphological variations in patients belonging to gender and various age groups in a Chinese population.

## Methods

### Sample selection

This study was approved by the Ethics Committee of Jinan Stomatological Hospital. The sampling process was preformed according to the guidelines proposed by Jorge et al. [23]. This was a retrospective study. All CBCT scans were acquired from a consecutive group of patients referred to Jinan Stomatological Hospital from January to December 2020. The CBCT images were mainly taken for the purposes of implant surgery, surgical removal of impacted teeth, orthodontic treatment, pre-surgical assessment of maxillofacial tumour and cysts or LeFort I osteotomy. The CBCT scans would be excluded if exist the following criteria: mandibular second molars with immature apices, with dental caries or defect, with endodontic treatment, with root resorption or calcification, with periapical lesions, with artificial crowns or post crowns, CBCT images with artifacts. Furthermore, in consideration of the side parameters, the images only with unilateral mandibular second molar were excluded from the study. After evaluation, 896 of 2683 CBCT scans (33%) were excluded.

The sample size was calculated based on single sample rate calculation formula:${\text{n}} = \left( {\frac{{Z_{\alpha } }}{\delta }} \right)^{2} \cdot \pi (1 - \pi )$. We calculated the maximal sample size using 95% confidence intervals (π = 50%, α = 0.05, δ = 0.05), which is 269. To exclude sampling bias, the sample size was enlarged to 1200. Stratified random sampling method was used to acquire the final study group. Finally, CBCT images of 1200 bilateral mandibular second molars from 600 patients (300 females and 300 males) were evaluated in this study. And all the patients were Chinese, aged from 15 to 69 years.

### Image acquisition

The CBCT images were taken using NewTom 5G system (Quantative Radiology s.r.l., Verona, Italy) operating at 110 kV and 16 mA with an exposure time of 5.4 s. The voxel size was 0.25 mm, and the slice thickness was 0.3 mm. Scans were performed by an experienced oral radiologist according to the manufacturer’s recommended protocol with the minimum exposure necessary for adequate image quality and, moreover, the lowest radiation dosage.

### Image evaluation

All CBCT images were evaluated retrospectively by two endodontists and one oral radiologist with at least 10 years of experience to reach a consensus using the CBCT device and its own software (NNT 4.6, QR Verona, Italy).

**Fig. 1:**
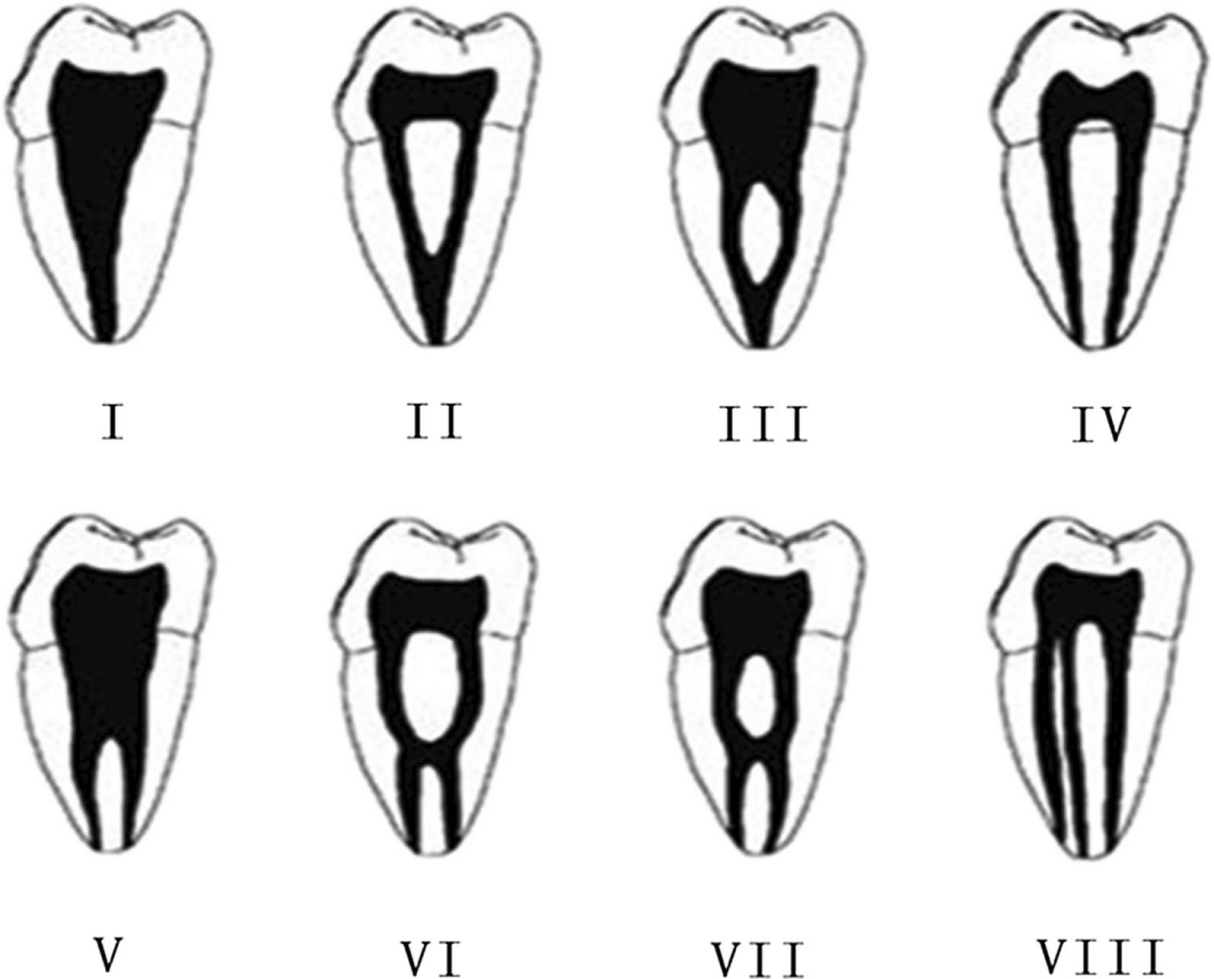
Vertucci’s classification of canal configuration

**Fig. 2:**
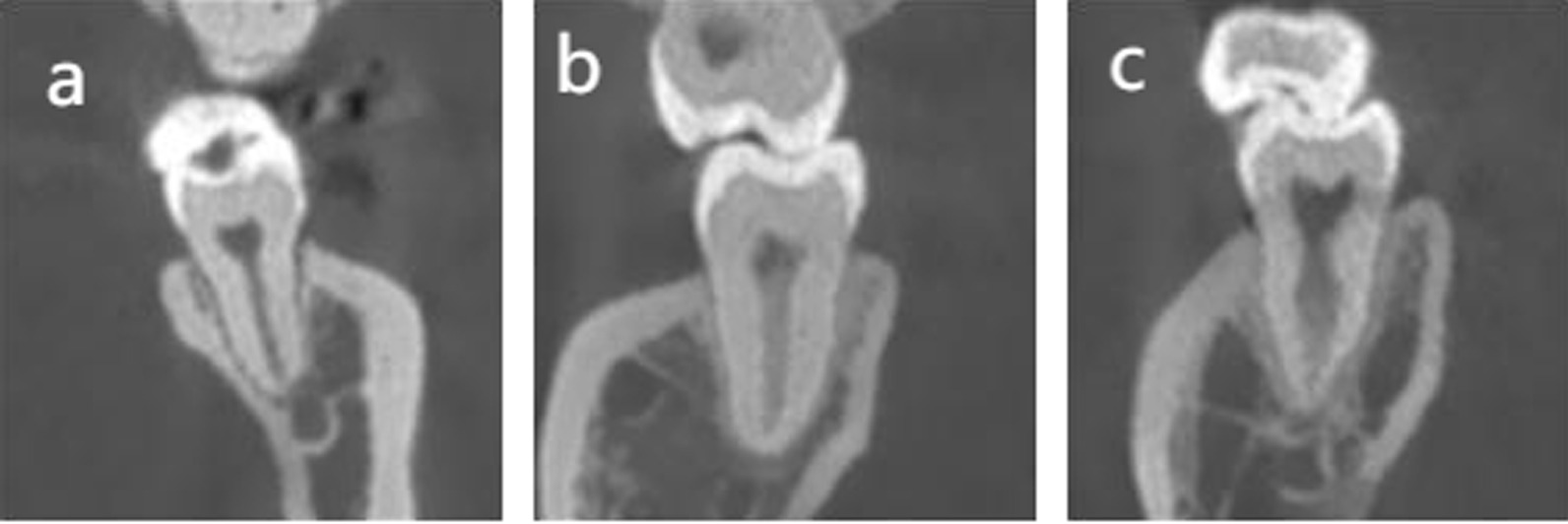
Coronal plan of Vertucci’s type I configuration. **a** a oval canal: the coronal plan of root canal is small and narrow and the cross-section is oval or round; **b** a flat canal: the coronal plan of root canal is large and wide and the cross-section is flat; **c** a expand canal: the middle part of the canal is expanded

**Fig. 3:**
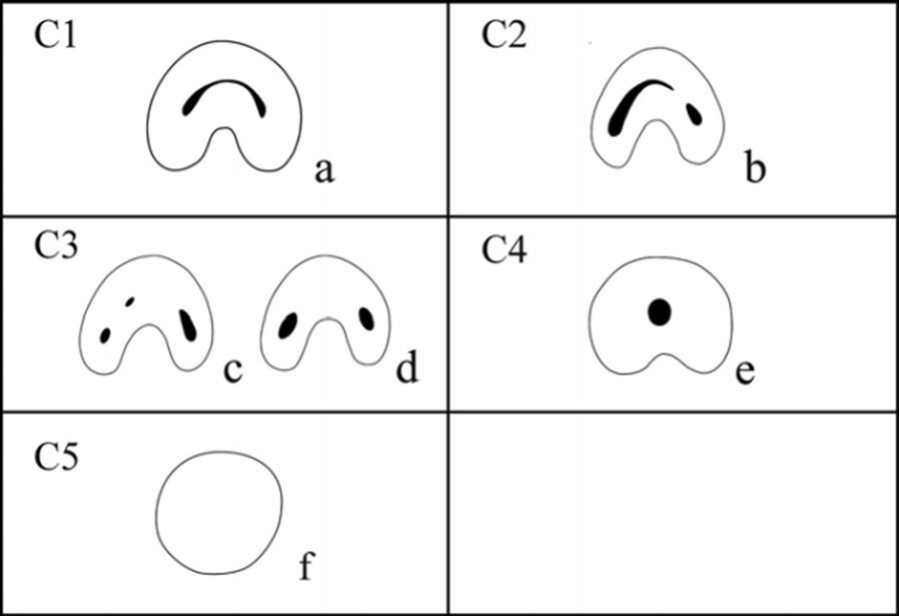
Classification of C-shaped canal configuration [24]. C1: continuous C-shaped canal (**a**); C2: the canal shape represents a semicolon resulting from discontinuation of the C outline (**b**); C3: three (**c**) or two (**d**) separate canals; C4: only one round- or oval-shaped canal in the cross-section (**e**); C5: no canal lumen could be observed (**f**)

The CBCT data were divided into 5 groups according to age: (1) 15–24 years; (2) 25–34 years; (3) 35–44 years; (4) 45–54 years; (5) ≥ 55 years; and 2 groups by gender: “females” and “males”. The CBCT images were evaluated in three plans (coronal, sagittal and axial) and the following information were recorded:

1. The number of roots and their morphology:
  O-shaped root: one single root and the cross-section is oval;
  C-shaped root: one single or fused root and the cross-section is C-shaped;
  O-C-shaped root: one single root in which the top part cross-section is oval and the bottom part is C-shaped.
2. The number of canals per root;
3. The root canal configuration in each root for two-rooted teeth classified using Vertucci’s classification [12] (Fig. 1); the internal configuration of one root canal (type I) was analysed from coronal plan, by which the type I configuration was classified into three types: (i) a oval canal (Fig. 2a) (the coronal plan of root canal is small and narrow and the cross-section is oval or round); (ii) a flat canal (Fig. 2b) (the coronal plan of root canal is large and wide and the cross-section is flat); (iii) a expand canal (the middle part of the canal is expanded) (Fig. 2c).
4. The frequency and configuration of C-shaped canals by gender, age and tooth position (left side and right side);

The mandibular second molars definited as having a C-shaped canal system had to exhibit all the following three features:

- fused roots;
- a longitudinal groove on lingual or buccal surface of the root;
- at least one cross-section of the canal belongs to the C1, C2, or C3 configuration by Fan et al. [24] (Fig. 3)
  Category I (C1): the root has a continuous C-shaped cross-section with no separation or division;
  Category II (C2): the root has a discontinuous C-shaped cross-section with at least one arc that is no less than 60°;
  Category III (C3): the root has 2–3 separate canals and no arc is more than 60°;
  Category IV (C4): only one round or oval canal in that crosssection;
  Category V (C5): no canal lumen could be observed.

5. The frequency of special Taurodontism canal configuration.

**Fig. 4:**
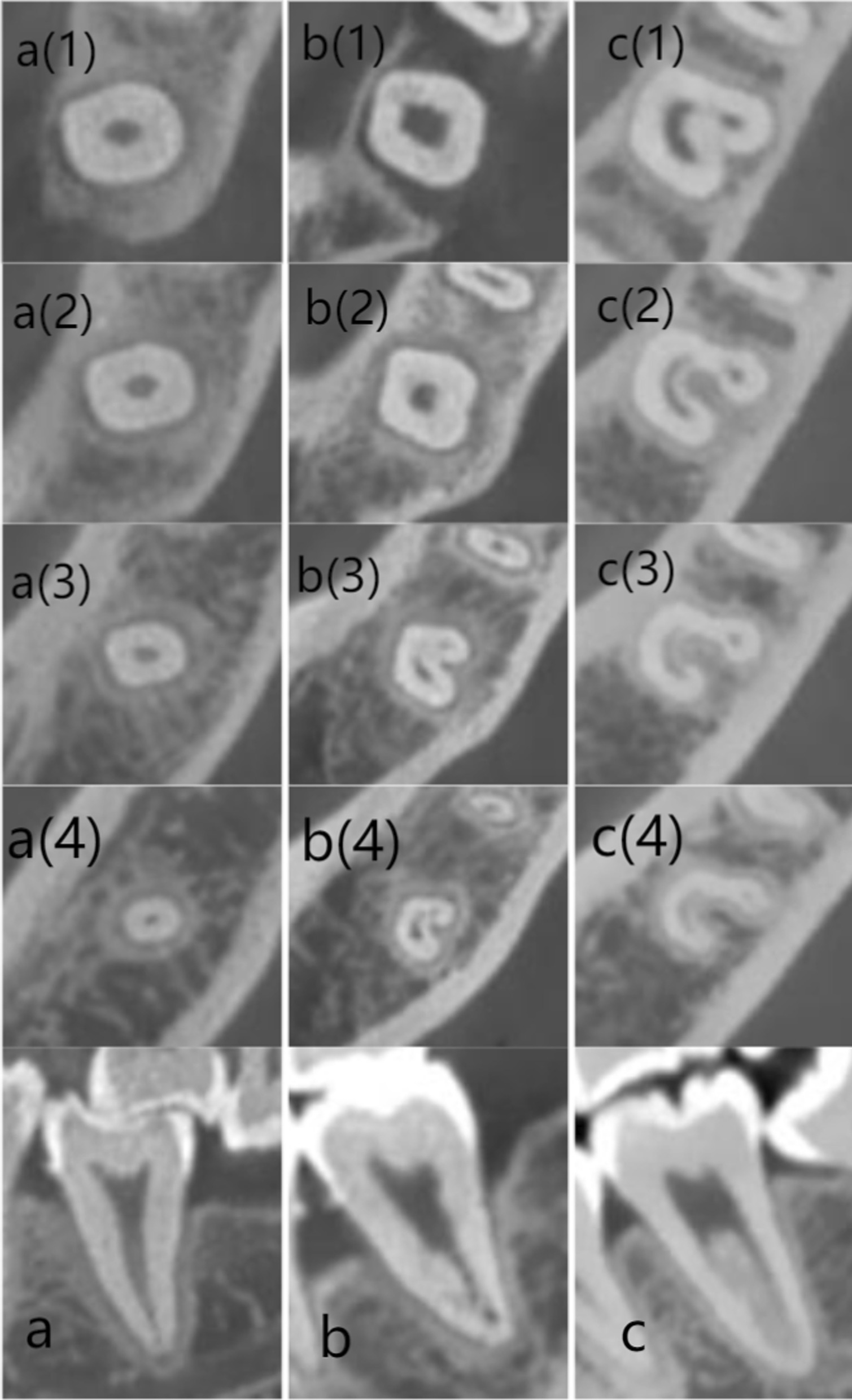
Root and canal configuration of mandibular second molars with one root at different root levels. **a** one O-shaped root with one single root canal. **b** One O-C-shaped root with Taurodontism canal. **c** One C-shaped root with C-shaped root canal. (1) Canal orififice. (2) Coronal level. (3) Middle level. (4) Apical level

In tooth with Taurodontism configuration, the pulp chamber is greatly enlarged, characterized by displacement of the furcation to the apical area [25] (Fig. 4b). In the enlarged part is a single oval canal while from the furcation to the apical area is unusual and irregular C-shaped canal.

The number and configuration of roots, the number of root canals, the root canal configuration and the correlations with gender, age and tooth position (left side and right side), the unilateral and bilateral occurrence rates of C-shaped root canal systems were analyzed.

Statistically significant differences were evaluated using the chi-square test with SPSS 16.0 for Windows (SPSS, Chicago, IL). *P* value < 0.05 was considered statistically significant.

### Reliability

To determine the intrarater and interrater reliability, every observer evaluated the same CBCT images of 60 selected mandibular second molars twice with a 2-week interval between assessments.The Cohen kappa test was used to analyse the reliability. The observers were considered reliable if the kappa value was equal or superior 0.8. In this study, the kappa values were 0.928 /0.856(intrarater) and 0.853 (interrater), reflected very high agreement.

## Results

### Roots morphology

**Fig. 5:**
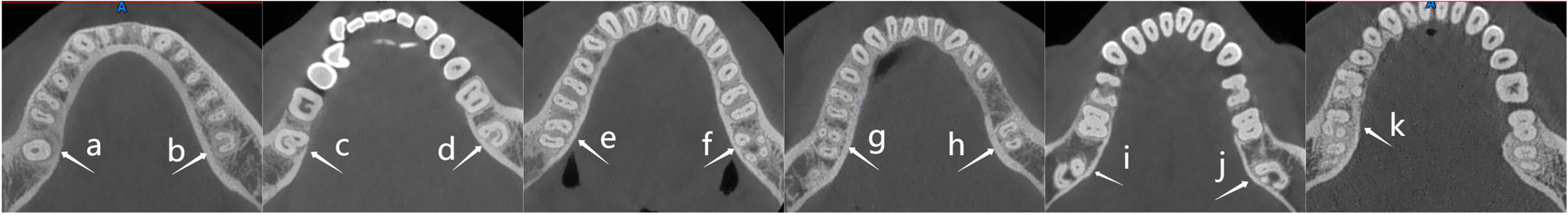
Root number and configuration of mandibular second molars. Arrows denote the examined teeth. **a** O-shaped root. **b**–**d** C-shaped root. **e**, **h** Two seperate roots: one mesial root and one distal root. **f** Four roots: two mesial roots and two distal roots. **g** Three roots: two mesial roots and one distal root. **i** C + ML root:Mesiolingual root in combination with C-shaped root. **j** C + L root:mid-lingual rootin combination with C-shaped root. **k** C + DL root: Distolingual root in combination with C-shaped root

The number of roots were listed in Table 1 and the root morphology were shown in Figs. 4 and 5. Of the 1200 mandibular second molars, 61% had two separate roots located mesiodistally, followed by one C-shaped root (35.6%). Twenty teeth (1.66%) were detected have one O-shaped root (Fig. 4a) and 5 teeth (0.41%) were detected have one O-C-shaped root (Fig. 4b). The prevalence of three roots and four roots were 0.16% (2 teeth) and 0.25% (3 teeth). Total 10 teeth (0.83%) have an additional root [26]: one C-shaped root with a mesiolingual (4 teeth, 0.33%) or distolingual (3 teeth, 0.25%) or mid-lingual root (3 teeth, 0.25%).

**Table 1:** Number and frequency of roots in mandibular second molars by gender and age

Root numbers n (%)	Gender		Age					Total of all
	Female (n = 600)	Male (n = 600)	15–24 (n = 400)	25–34 (n = 220)	35–44 (n = 180)	45–54 (n = 260)	≥ 55 (n = 140)	N = 1200
1-O-shaped root^a^	14 (2.33)	6 (1.0)	7 (1.75)	4 (1.81)	3 (1.66)	6 (2.3)	–	20 (1.66)
1-C-shaped root^b^	255 (42.5)*	172 (28.6)*	155 (38.75)^§^	64 (29.1)^§^	58 (32.2)^§^	97 (37.3)^§^	53 (37.8)^§^	427 (35.6)
1-O-C-shaped root^c^	1 (0.16)	4 (0.66)	1 (0.25)	4 (1.81)	–	–	–	5 (0.41)
C + ML root^d^	3 (0.5)	1 (0.16)	2 (0.5)	–	1 (0.55)	1 (0.38)	–	4 (0.33)
C + DL root^e^	1 (0.16)	2 (0.33)	–	–	1 (0.55)	2 (0.76)	–	3 (0.25)
C + L root^f^	3 (0.5)	–	2 (0.5)	–	–	1 (0.38)	–	3 (0.25)
Two roots^g^	321 (53.5)**	412 (68.6)**	232 (58.0)^§§^	146 (63.6)^§§^	116 (64.4)^§§^	152 (58.4)^§§^	87 (62.1)^§§^	733 (61.0)
Three roots^h^	–	2 (0.33)	1 (0.25)	–	–	1 (0.38)	–	2 (0.16)
Four roots^i^	2 (0.33)	1 (0.16)	–	2 (0.9)	1 (0.55)	–	–	3 (0.25)

^a^One single root and the cross-section is oval

^b^One single or fused root and the cross-section is C-shaped

^c^One single root in which the top part cross-section is oval and the bottom part is C-shaped

^d^One C-shaped root with an additional mesiolingual root

^e^One C-shaped root with an additional distolingual root

^f^One C-shaped root with an additional mid- lingual root

^g^One mesial root and one distal root

^h^Two mesial roots and one distal root

^i^Two mesial roots and two distal root

By chi-square test: *P = 0.000; **P = 0.000; ^§^*P* = 0.107; ^§§^*P* = 0.210

Two-rooted mandibular second molars were significantly frequent in males when compared with the females: 68.6% vs 53.5% (*P* = 0.000). On the contrary, C-shaped root morphology were significantly frequent in females than males: 42.5% vs 28.6% (*P* = 0.000). The prevalence of two-rooted and C-shaped rooted mandibular second molars had no statistical difference between each age groups (*P* = 0.107, 0.210).

### Root canal configuration based on Vertucci’s classification

The number of root canals was displayed in Table 2. The 45.3% teeth had three canals in two-rooted mandibular second molars, 13.4% and 2.33% had two canals and four canals respectively. Thirteen (1.08%) teeth had only one single canal (Fig. 4a). Nine (0.75%) teeth had the special Taurodontism canal (Fig. 4b) [25]. Total of 430 (35.8%) teeth had C-shaped canal (Fig. 4c). In three-rooted and four-rooted teeth, every root exhibited type I canal configuration. As for the teeth with additional roots, extra root exhibited type I canal configuration and the other root exhibited C-shaped root canal.

**Table 2:** Number and frequency of root canals in mandibular second molars (N = 1200)

	1 canal	2 canals	3 canals	4 canals	C-shaped canal	Taurodontism canal	Total
1 root	13 (1.08)	–	–	–	430 (35.8)	9 (0.75)	452
2 roots	–	161 (13.4)	544 (45.3)	28 (2.33)	–	–	733
3 roots	–	–	2 (0.16)	–	–	–	2
4 roots	–	–	–	3 (0.25)	–	–	3

Tables 3 and 4 showed the canal configuration of mesial and distal root in two-rooted mandibular second molars. The mesial root showed a Vertucci type II configuration in 28.9% cases followed by type IV (24.4%), type I (22.5%), type III (14.6%) and type V (7.9%). The type II and type IV configurations showed significant difference between the gender groups (*P* = 0.031 and 0.005, respectively), and did not differ in age groups (*P* = 0.524 and 0.266, respectively).

**Table 3:** Frequency distribution of root canal configurations in mesial root for 733 two-rooted mandibular second molars

Canal configuration N (%)	Gender		Age					Total of all
	Female (n = 321)	Male (n = 412)	15–24 (n = 232)	25–34 (n = 146)	35–44 (n = 116)	45–54 (n = 152)	≥ 55 (n = 87)	733
Type I, oval canal	7 (2.18)	4 (0.97)	–	–	2 (1.72)	8 (5.26)	1 (1.14)	11 (1.5)
Type I, flat canal	50 (15.5)	59 (14.3)	42 (18.1)	25 (17.1)	13 (11.2)	16 (10.5)	13 (14.9)	109 (14.8)
Type I, expand canal	19 (5.9)	26 (6.3)	21 (9.0)	12 (8.2)	4 (3.44)	6 (3.94)	2 (2.29)	45 (6.13)
Type I, Total	76 (23.6)	89 (21.6)	63 (27.1)	37 (25.3)	19 (16.3)	30 (19.7)	16 (18.3)	165 (22.5)
Type II, 2-1 canal	106 (33.0)*	106 (25.7)*	64 (27.5)^§^	48 (32.8)^§^	38 (32.7)^§^	40 (26.3)^§^	22 (25.2)^§^	212 (28.9)
Type III, 1-2-1 canal	45 (14.0)	62 (15.0)	31 (13.3)	21 (14.3)	23 (19.8)	20 (13.1)	12 (13.7)	107 (14.6)
Type IV, 2-2 canal	62 (19.3)**	117 (28.4)**	53 (22.8)^§§^	28 (19.1)^§§^	30 (25.8)^§§^	41 (26.9)^§§^	27 (31)^§§^	179 (24.4)
Type V, 1-2 canal	24 (7.47)	34 (8.2)	19 (8.2)	8 (5.4)	6 (5.17)	19 (12.5)	6 (6.89)	58 (7.9)
Type VI, 2-1-2 canal	4 (1.24)	–	–	1 (0.68)	–	1 (0.65)	2 (2.29)	4 (0.54)
Type VII, 1-2-1-2 canal	2 (0.62)	4 (0.97)	1 (0.43)	2 (1.36)	–	1 (0.65)	2 (2.29)	6 (0.8)
Type VIII, 3 canal	–	–	–	–	–	–	–	
Other 1-3-1 canal	1 (0.31)	–	1 (0.43)	–	–	–	–	1 (0.13)
Other 3-1 canal	1 (0.31)	–	–	1 (0.68)	–	–	–	1 (0.13)

By chi-square test: *P = 0.031; **P = 0.005; ^§^*P* = 0.524; ^§§^*P* = 0.266

**Table 4:** Frequency distribution of root canal configurations in distal root for 733 two-rooted mandibular second molars

Canal configuration N (%)	Gender		Age					Total of all
	Female (n = 321)	Male (n = 412)	15–24 (n = 232)	25–34 (n = 146)	35–44 (n = 116)	45–54 (n = 152)	≥ 55 (n = 87)	733
Type I, oval canal^a^	138 (42.9)	187 (45.3)	66 (28.4)^§^	51 (34.9)^§^	52 (44.8)^§^	95 (62.5)^§^	61 (70.1)^§^	325 (44.3)
Type I, flat canal^b^	87 (27.1)	135 (32.7)	104(44.8)^§§^	49 (33.5)^§§^	36 (31.0)^§§^	27 (17.7)^§§^	6 (6.89)^§§^	222 (30.3)
Type I, expand canal^c^	79 (24.6)	75 (18.2)	52 (22.4)	39 (26.7)	22 (18.9)	26 (17.1)	15 (17.2)	154 (21)
Type I, Total	304(94.7)	397 (96.3)	222(95.6)	139(95.2)	110(94.8)	148(97.3)	82(94.2)	701 (95.6)
Type II, 2–1 canal	-	5 (1.21)	-	4 (2.73)	-	1 (0.65)	-	5 (0.68)
Type III,1–2-1 cnal	7 (2.18)	5 (1.21)	5 (2.15)	-	4 (3.44)	-	3 (3.44)	12 (1.6)
Type V,1–2 canal	10 (3.11)	5 (1.21)	5 (2.15)	3 (2.1)	2 (1.72)	3 (1.97)	2 (2.29)	15 (2)

^a^A small and narrow canal

^b^A large and wide canal

^c^The middle part of the canal is expanded

By chi-square test: ^§^*P* = 0.000; ^§§^*P* = 0.000

The distal root showed a remarkably high Vertucci type I frequency (95.6%), in which the oval canal was the most prevalent type (44.3%) and was positively correlated with age. The prevalence of type I -flat canal decreased with age growing(44.8%,33.5%,31%, 17.7%, 6.89%). The prevalence of oval canal and flat canal of Vertucci type I in distal root were significantly different in different age groups (*P* = 0.000).The occurrence of type II, type III, and type V was relatively rare (0.68%, 1.6%, and 2%, respectively).

### C-shaped root canal

Tables 5 and 6 showed the frequency and configurations of C-shaped root canal by gender, age and position. Total of 430 teeth (35.8%) had C-shaped root canals. The prevalence of C-shaped root canal systems was significantly higher in females (42.5%) than in males (29.1%) (*P* = 0.000), and did not differ with age (*P* = 0.126). It should be noted that the lowest percentage were in 25–34 age group (29.5%).

**Table 5:** Number frequency and root canal configurations at orifice cross-sectional lever of C-shaped root canals in mandibular second molars by gender and age (N = 430)

Canal configuration n (%)	Gender		Age					Total
	Female (n = 600)	Male (n = 600)	15–24 (n = 400)	25–34 (n = 220)	35–44 (n = 180)	45–54 (n = 260)	≥ 55 (n = 140)	
C1 canal	55 (21.6)	44 (25.1)	49 (31.4)	14 (21.5)	12 (20.6)	20 (20.4)	4 (7.54)	99 (23.1)
C2 canal	87 (34.1)	64 (36.6)	60 (38.4)	24 (36.9)	14 (24.1)	36 (36.7)	17 (32.1)	151 (35.1)
C3 canal	113 (44.3)	67 (38.3)	47 (30.1)	27 (41.5)	32 (55.1)	42 (42.8)	32 (60.3)	180 (41.8)
Total of C-shaped root canals	255* (42.5)	175* (29.1)	156^§^ (39)	65^§^ (29.5)	58^§^(32.2)	98^§^(37.7)	53^§^(37.8)	430

By chi-square test: *P = 0.000; ^§^ *P* = 0.126

**Table 6:** Prevalence of unilateral and bilateral occurrences of 430 mandibular second molars with C-shaped canals among 257 patients

	Unilateral			Bilateral n (%)
	Left n(%)	Right n(%)	Total n(%)	
Total of patient (n = 257)	43 (16.7)*	41 (16)*	84 (32.7)^§^	173 (67.3)^§^
Total of teeth (n = 430)	43 (10)	41 (9.5)	84 (19.5)	346 (80.4)

By chi-square test: *P = 0.758; ^§^*P* = 0.000

The prevalence of bilateral C-shaped root canal was 67.3% in 257 patients and 80.4% in 430 teeth. This concurrent appearance was statistically significant (*P* = 0.000). And the prevalence was not different in left and right side (*P* = 0.758).

Table 5 showed the configurations of C-shaped roots at orifice cross-sectional level by gender and age. The most frequent C-shaped root canal configuration is C3 (41.8%) and the lowest is C1 (23.1%) at the orifice level. And as the age increasing, the C1 configuration is lower(31.4% in 15–25 age group, 21.5% in 25–34 age group, 20.6% in 35–44 age group, 20.4% in 45–54 age group, 7.54% in ≥ 55 age group).

## Discussion

Successful root canal treatment depends on good knowledge of the root canal anatomy. A lack of knowledge about root canal systems may lead to endodontic treatment failure. The mandibular second molars have a variation in root and canal morphology and numbers which increased the difficulty of clinical treatment. This study comprised a detailed investigation of root and canal morphology of mandibular second molars in a Chinese population using CBCT by gender and age.

The most commonly observed root morphology for the mandibular second molars was 2 separate roots with three canals. In this study the prevalence of two-rooted mandibular second molars is 61% and have a significant difference by gender (68.6% of males, 53.5% of females, *P* = 0.000). The result is lower than the other report on root canal morphology of mandibular second molars in the Chinese population [7]. The afore-mentioned study reported two-rooted mandibular second molars in 76% of the specimens studied. Such a difference could be attributed to variations in the sample size:157 molars vs 1200 molars. Moreover the result is similar to the prevalence in Korean population (58%) and Asian ethnic group (53.8%) [8, 27] and lower than the prevalence in Indian population (79.35%), in a south-eastern Turkish population (90%) and in White ethnic group (83.1%) [4, 6, 27]. The prevalence of three canals in two roots is 45.3% in this study and is lower than other populations, which are Indian (53.5%), Moscow (82.2%), Brazilian (54%) and Turkish (72.8%) populations [4, 9, 28, 29].Table 7 shows the prevalence of root and canal configurations in mandibular second molars in each region.

**Table 7:** Prevalence of root and canal configurations in mandibular second molars in previous studies by CBCT

References	Region/race	Sample size	Prevalence of two-rooted teeth (%)	Prevalence of three canals in two-rooted teeth (%)	Prevalence of C-shaped root canal (%)
This study	Chinese	1200	61	45.3	35.8
Ajinkya et al. [4]	Indian	983	79.35	53.5	13.12
Nur et al. [6]	South-eastern Turkish	1165	90	–	–
Zhang et al. [7]	Chinese	389	76	46	29
Kim et al. [8]	Korean	1920	58	81.6	40
Razumova et al. [9]	Moscow	398	99.5	82.2	-
Martins et al. [27]	Asian	240	53.8	–	–
	White	687	83.1	–	–
Silva et al. [28]	Brazilian	226	86	54	3.5
Demirbuga et al. [29]	Turkish	925	85.4	72.8	4.1

The mesial roots of two-rooted second molars predominantly exhibited two canals.The most common root canal configuration in the mesial root canal was type II (28.9%) followed by type IV (24.4%) (19.3% of females and 28.4% of males). However, teeth with a complex root canal system (type VI, VII, and VIII) are difficult to diagnose and treat with subsequent cleaning, shaping, and obturation. Knowing the presence of these types of root canal systems can avoid the omission of root canals and attribute to the successful root canal treatment.

Root canal anatomy is indeed to change over the years because of physiological or pathological factors like the deposition of secondary dentine which starts to form once the tooth erupts and is in occlusion [20]. Young patients tend to have large single canals and pulp chambers [30] while older patients tend to have narrower root canals [31]. The study by Martins et al. revealed a global tendency of a greater Vertucci Type I prevalence in younger patients [10]. In the present study, a subtle decrease presence of Vertucci Type I was noted in the mesial root as aging (Table 3).The distal roots predominantly exhibited a single canal type I. While as the age increasing, the prevalence of oval canal (small and narrow) in mesial and distal roots were increased and on the contrary the prevalence of flat canal (large and wide) were decreased. For the middle narrow part of flat canal and the expanded part of expand canal, they are easy to be unprepared during endodontic treatment which corresponding lead to the treatment failure. Therefore when deal with the root canal of young people the complete instrumentation is essential even though it is a single root canal.

Mandibular second molars generally have two roots, mesial and distal. However, due to differences in ethnic origin or differences in tooth anatomy, an additional third root may be found, and the first sample was found by Carabelli in 1844 [32]. The prevalence of additional root in mandibular second molars was found to be 1.2% by Zhang [7], 0.7% by Song [33], 0.6% by Suayip [25] and 0.83% in our study. All those studies were performed by CBCT images. It is difficult to detect the third roots with conventional radiography because the images of the roots overlap and the roots themselves are generally shorter and more curved than other roots [34]. When root canal treatment is performed, special attention to the extra roots is necessary, entrance cavities should be opened in trapezoidal figure to ease access to canal openings and to help the localization of extra canal orifices. Clearly, negotiate and the failure to locate the additional root canal may result in treatment failure.

In this study mandibular second molars with three roots were detected in two teeth (0.16%) and four roots were detected in three teeth (0.25%). In another report in mandibular second molars in the Chinese population also showed two teeth (1.27%) exhibited three roots [7]. In Asian Ethnic Group the prevalence of three-rooted teeth was 0.8% [26]. In other studies, the data was ranged from 0.7 to 7.35% in different populations. As for four roots, only one study in a Turkish Cypriot population reported one same sample [5].

In the total of 1200 mandibular second molars of this study,5 teeth (0.41%) had one single root in which the top part cross-section is oval and the bottom part is C-shaped (Fig. 4, b) and 20 teeth (1.66%) had one single oval-shaped root (Fig. 4, a). The prevalence of the former root configuration has not been previously reported. The unusual O-shaped roots was previously documented in 1.6% Turkish population [5] and 0.1% in Korean population [8].

Of the single-rooted teeth, 9 teeth (0.75%) exhibited an unusual Taurodontism configuration in which the pulp chamber is greatly enlarged, characterized by displacement of the furcation to the apical area [25] (Fig. 4b). Taurodontism could be detected with conventional radiography generally. However the internal root canal configuration is complicated. In the enlarged part is a single oval canal while from the furcation to the apical area is unusual and irregular C-shaped canal. These root canal variation provided a significant challenge to clinician.

Previous studies revealed that the prevalence of C-shaped configurations in mandibular second molars had a ethnic difference which is higher in East Asian populations (29–44%), when compared with those of Western countries (3.6–9.3%) [7, 8, 14, 27, 28]. In this study, the prevalence of C-shaped root canal is 35.8% which is consistent with present study results. What’s more, the study among the global sample about C-shaped root canals improved that female patients showed higher prevalence in every single region [14]. In another study in an Iraqi Subpopulation by Kazhan et al. [35] also showed a significantly higher prevalence of C-shaped root canal in females (23%) than males (10.4%) and the prevalence did not differ with age or tooth position. The same results is found in this study: 42.5% of females and 29.1% of males, being statistically significant. In a previous study in the United States [36], the possibility of bilateral occurrence of C-shaped canals was 70%. In another study, the data was improved to 82% in a Korean subpopulation [8]. In this study, C-shaped canals were found to demonstrate symmetric distribution in 80.4% of the samples. This has very high clinical relevance in endodontic therapy.

The root canal anatomy of C-shaped mandibular molars is highly variable. Many studies reported that C-shaped canals can vary in number and shape along the length of the root [18, 24, 37]. The presence of fins and isthmus make it more difficult to shape, clean and fill the root canal system. Previous studies had shown that half the canal walls are untouched by instruments used for root canal preparation [38–40]. Thus suitable instruments in combination with alternative canal cleaning techniques such as the use of passive ultrasonic irrigation are recommended when deal with C-shaped canals.

In summary, the reported data may help clinicians understand the variations in the root canal morphology of mandibular second molars. CBCT scanning can allow practitioners to detect the variations and fully evaluate the anatomy from 3-dimensional plane, it should be considered as the gold standard in diagnosis and treatment. However, considering the higher radiation and charge of CBCT imaging compared with periapical radiography,it could not be used as a routine examination. When variations were detected in traditional films such as Taurodontism and extra root, it is necessary to adopt CBCT for endodontic treatment. At the same time, effective preventive measures should be taken to reduce overexposure to radiation.

The current study showed the root and canal morphology of the mandibular second molars in Chinese population which provided some theoretical guidance for endodontic treatment. However there are also some limitations in this study. Root canal anatomy is changed over the years because of physiological or pathological factors like the deposition of secondary dentine, so a greater sample size of every age group may improve more information between age and root canal morphology. As we all know, there are 56 nations in Chinese. The ethnic factors were not considered in this study. Further research could be done to provide more information of the minority nations especially in remote regions.

## Conclusions

The root and canal anatomy of 1200 Chinese mandibular second molars was investigated using CBCT. The most commonly observed root morphology for the mandibular second molars was 2 separate roots with three canals. The prevalence of C-shaped root canal is 35.8% and is more higher in females than in males. Knowing the complex root and canal morphology play an important role in endodontic treatment of mandibular second molars. CBCT is a potentially effective tool in the endodontic diagnosis and treatment.

## Data Availability

The dataset used and/or analyzed during the current study are available from the corresponding author on reasonable request.

## Abbreviation

CBCT: Cone-beam Computed Tomography
